# Reviewed article: Alberti AM, Silva PBE, Azambuja FS, Godoy L, Concolatto FP, Lech GE, Stein AT. Epidemiological profile of surgical treatment of lung cancer in Brazil: retrospective analysis (2014-2023). Epidemiol Serv Saude. 2025:34;e20240427

**DOI:** 10.1590/S2237-96222025v34e20240427.a

**Published:** 2025-08-04

**Authors:** Maryana Carmello da Costa

**Affiliations:** FOUSP

**Table:** 

Rating	Excellent	Good	Average	Below average	Poor
Interest	✓				
Quality		✓			
Originality	✓				
Overall		✓			

**Table:** 

Questionnaire	Yes	No	Not applicable
Does the manuscript contain new and significant information to justify publication?	✓		
Does the Abstract (Summary) clearly and accurately describe the content of the article?	✓		
Is the problem significant and concisely stated?	✓		
Are the methods described comprehensively?	✓		
Are the interpretations and conclusions justified by the results?	✓		
Is adequate reference made to other work in the field?	✓		

## Manuscript Structure

Length of article is: Adequate

Number of tables is: Adequate

Number of figures is: Adequate

Please state any conflict(s) of interest that you have in relation to the review of this paper (state “none” if this is not applicable): None

## Comments

Orientações em relação à escrita: “Os termos ‘sexo’ e ‘gênero’ foram utilizados como sinônimos e precisam ser revisados conforme as diretrizes SAGER. Substituir o ‘gênero’ do texto por ‘sexo’ ao longo do texto e nas ilustrações.

No resumo do manuscrito, na parte dos “métodos” é citado que foram analisadas hospitalizações, sexo, idade, etnia e óbitos porém esses dados aparecem incompletos no texto, na parte dos métodos, resultados e discussão. Sugere-se análise e correção.

Dê preferência à termos atuais, adequados e em português, substituir a palavra “insights”. 

